# Predicting diabetes mellitus using SMOTE and ensemble machine learning approach: The Henry Ford ExercIse Testing (FIT) project

**DOI:** 10.1371/journal.pone.0179805

**Published:** 2017-07-24

**Authors:** Manal Alghamdi, Mouaz Al-Mallah, Steven Keteyian, Clinton Brawner, Jonathan Ehrman, Sherif Sakr

**Affiliations:** 1 King Saud bin Abdulaziz University for Health Sciences, Riyadh, Saudi Arabia; 2 King Abdullah International Medical Research Center, Riyadh, Saudia Arabia; 3 Heart and Vascular Institute, Henry Ford Hospital System, Detroit, MI, United States of America; Harbin Institute of Technology Shenzhen Graduate School, CHINA

## Abstract

Machine learning is becoming a popular and important approach in the field of medical research. In this study, we investigate the relative performance of various machine learning methods such as Decision Tree, Naïve Bayes, Logistic Regression, Logistic Model Tree and Random Forests for predicting incident diabetes using medical records of cardiorespiratory fitness. In addition, we apply different techniques to uncover potential predictors of diabetes. This FIT project study used data of 32,555 patients who are free of any known coronary artery disease or heart failure who underwent clinician-referred exercise treadmill stress testing at Henry Ford Health Systems between 1991 and 2009 and had a complete 5-year follow-up. At the completion of the fifth year, 5,099 of those patients have developed diabetes. The dataset contained 62 attributes classified into four categories: demographic characteristics, disease history, medication use history, and stress test vital signs. We developed an Ensembling-based predictive model using 13 attributes that were selected based on their clinical importance, Multiple Linear Regression, and Information Gain Ranking methods. The negative effect of the imbalance class of the constructed model was handled by Synthetic Minority Oversampling Technique (SMOTE). The overall performance of the predictive model classifier was improved by the Ensemble machine learning approach using the Vote method with three Decision Trees (Naïve Bayes Tree, Random Forest, and Logistic Model Tree) and achieved high accuracy of prediction (AUC = 0.92). The study shows the potential of ensembling and SMOTE approaches for predicting incident diabetes using cardiorespiratory fitness data.

## Introduction

Over the last century, the prevalence of diabetes has been increasing dramatically with the aging population worldwide. Today, about 415 million people around the world have diabetes [1]. Globally, the projection of having diabetes will rise from one in 11 adults in 2015 to one in 10 adults by 2040 [1]. Diabetes is a significant contributor to increased mortality rates and thus reduction in life expectancy of elderly diabetic patients [2]. In 2015, diabetes was responsible for 4.5 million deaths around the world [1] and is projected to be the 7th leading cause of death in 2030. This epidemic disease is continuously escalating and a major economic burden on health care systems [1].

Known coronary artery disease was defined as an existing history of any of the following: myocardial infarction, coronary angioplasty, coronary artery bypass surgery, or documented obstructive CAD on angiogram. Heart failure was defined as a prior clinical diagnosis of systolic or diastolic heart failure (heart failure with reduced or preserved left ventricular function). Diabetes mellitus was defined as a prior clinical diagnosis of diabetes, use of anti-hyperglycemic medications including insulin, or an electronic medical record (EMR) or problem list-based diagnosis of diabetes [3].

Diabetes contributes significantly in increasing mortality and reducing life expectancy in elderly diabetic patients [4, 5]. The key problem is that patients who might develop diabetes are not aware of the associated high risks. Late or lack of diabetes diagnosis increases the chance of developing any disease due to chronic vascular complications [4, 5]. However, screening patients and detecting asymptomatic disease such as diabetes might help in delaying its progression and preventing its complications [2], controlling the treatment, and reducing the costs of this preventable disease in the health care system [4]. Furthermore, it is also beneficial for both public health and clinical practice in general [2]. Demographic characteristics such as age, sex and race are non-modifiable risk factors of diabetes. The association of these characteristics to diabetes has been explored in a number of studies and has proven their direct association to diabetes. Diabetes is more prevalent in men than in women [6–8] and increases with the increase of age [6, 9]; in 2015 there were about 199.5 million women who had diabetes in comparison to 215.2 million men [9]. Also, a systematic review by Alhyas et al. [7] has found that there is a significant relationship between incidents of diabetes and the increase in age of both sexes. Major risk factors of diabetes mellitus include obesity, physical inactivity, unhealthy diet [2, 9, 10], population growth, aging, urbanization [9], family history of diabetes, previous history of gestational diabetes and ethnicity groups [10].

Machine learning methods are gaining increasing momentum and attracting a lot of attention in the field of medical research [11]. They have shown their capabilities to effectively deal with large numbers of variables while producing powerful predictive models. They also embed variable selection mechanisms which can detect complex relationships in the data. Supervised classification techniques [12] are popular machine learning methods that aim to explain the dependent variable in terms of the independent variables. The aim of this study is to take advantage of the unique opportunity provided by our access to a large and rich clinical research dataset collected by the The Henry Ford ExercIse Tesing (FIT) project [13] and using it to investigate the relative performance of various machine learning classification methods such as Decision Tree (DT), Naïve Bayes (NB), Logistic Regression (LR), Logistic Model Tree (LMT) and Random Forests (RF) for predicting incident diabetes using medical records of cardiorespiratory fitness. In addition, we apply different techniques to uncover potential predictors of diabetes using the available large set of dataset attributes.

## Materials and methods

### Henry Ford FIT dataset

The dataset was collected from patients who underwent treadmill stress testing by physician referrals at Henry Ford Affiliated Hospitals in metropolitan Detroit, MI in the U.S. The FIT Project data has been obtained from the electronic medical records, administrative databases, and the linked claim files and death registry of the hospital [13]. Study participants underwent routine clinical treadmill exercise stress testing using the standard Bruce protocol between January 1st, 1991 and May 28th, 2009. The day the treadmill test was performed served as the baseline for this study. The exercise stress test would be terminated by the supervising clinician if the patient had exercise-limiting chest pain, shortness of breath, or other limiting symptoms independent of the achieved heart rate. Furthermore, testing could also be terminated early at the discretion of the supervising clinician for significant arrhythmias, abnormal hemodynamic responses, diagnostic ST-segment changes, or if the participant was unwilling or unable to continue.

This FIT project study used data of 32,555 patients free of known coronary artery disease or heart failure who underwent clinician-referred exercise treadmill stress testing at Henry Ford Health Systems Between 1991 and 2009 and had a complete 5-year follow-up. The dataset contained four categories: demographic characteristics, disease history, medication use history, and exercise test data for 62 attributes. At the completion of the fifth year, 5,099 of those patients have developed diabetes. Resting heart rate and blood pressure were measured in the seated position prior to treadmill testing. The percent of maximal heart rate achieved was based on the age-predicted maximal heart rate formula: 220—age. Cardiorespiratory fitness, expressed in metabolic equivalents (METs), was based on the workload derived from the maximal speed and grade achieved during the total treadmill time. MET results were categorized into 4 groups based on distribution of the data as follows: < 6, 6–9, 10–11, ≥ 12 METs. For detailed description of the final dataset, see S1 Appendix.

### Data preprocessing

#### Data discretization

All binary attributes were transformed to nominal with Yes and No values including the label class (diabetic/non-diabetic). Also, all continuous numeric attributes were discretized by the unsupervised discretization filter using different bins range precision depending on the type of the attribute.

#### Feature selection

Feature selection is the main process of data dimensionality reduction; selecting subset of features that contribute significantly to the target class improves the overall prediction performance of the classifier, reduces the length of the process as well as the cost of computation [14]. Also, it clarifies the underlying process that generates the data [15]. In this study, the first group of attributes (G1) consisted of 26 attributes that were selected manually based on their clinical importance in the domain. Then, the SPSS statistical software was used to find the significant p-value for each attribute in relation to the target class by using Multiple Linear Regression (MLR). Furthermore, these 26 attributes were evaluated by the Attribute Evaluator in the WEKA software (http://www.cs.waikato.ac.nz/ml/weka/) using the Information Gain Technique (Entropy) [16]. Table 1 shows the significance rank of these attributes where the Age is ranked the highest in the list while Calcium Channel Blocker Medication is ranked the least. The second group of attributes (G2), highlighted in **bold** font in Table 1, have been deduced from the attributes of (G1). The selection was based on the highest ranked attributes that scored **0.01** or more (See Table 1). G2 included 13 attributes which are half the number of the first group (G1).

**Table 1:** Ranking of the dataset attributes based on their Information Gain (IG).

Attribute	IG Rank
**1. Age**	**0.433152**
**2. Resting Heart Rate**	**0.432196**
**3. Metabolic Equivalent**	**0.336157**
**4. Resting Systolic Blood Pressure**	**0.289812**
**5. Resting Diastolic Blood Pressure**	**0.28533**
**6. Sedentary Lifestyle**	**0.195819**
**7. Black**	**0.190509**
**8. Obesity**	**0.16529**
**9. Hypertension**	**0.100523**
**10. % HR Achieved**	**0.041825**
**11. Hyperlipidemia**	**0.028451**
**12. Aspirin**	**0.014868**
**13. Family History of Premature Coronary Artery Disease**	**0.01158**
14. Coronary Artery Disease	0.009904
15. Nitrate Use	0.009702
16. Diuretic Use	0.006716
17. Beta Blocker Use	0.006402
18. Sex	0.005626
19. Smoking	0.004923
20. Plavix Use	0.009904
21. Angiotensin	0.001397
22. Angiotensin Receptor Blockers Use	0.001154
23. Other Hypertension Medication Use	0.001132
24. Prior Cerebrovascular Accident	0.0008
25. Congestive Heart Failure	0.000777
26. Calcium Channel Blocker	0.000242

### Machine learning classification models

Classification technique is one of the most important machine learning prediction models [17]. Classification is described as the process of systematic arrangement of objects in groups or categories according to observed similarities. Many studies for predicting diabetes have used this type of classification, and this algorithm model has been proven to be highly effective in our study as well [18–20].

J48 [21] is a decision tree classification algorithm that generates a mapping tree that includes attributes nodes linked by two or more sub-trees, leaves, or other decision nodes. When building the classifier for this study, pruning was used to avoid the over-fitting problem. J48 uses the post-pruning approach that removes branches when the model tree is completed. Naïve Bayes Tree is another decision tree algorithm that generates a decision tree with naïve Bayes classifiers [22] at the leaves levels.

Logistic Regression (LR) [23] is a statistical classifier that provides the probability for predicting the labeled class of categorical type by using a number of attributes. The prediction model classifier measures the relationship between the attributes and the labeled class. Naïve Bayes (NB) [24] provides a probability based on the theorem of Bayes which is one of the Bayesian network algorithms that is well-known for its simplicity and good performance. It is built with the assumption of conditional independency between the attributes. The model does not require any iterative parameter estimation; therefore, it is very suitable for large datasets.

The Logistic Model Tree (LMT) algorithm [23–26] is a supervised training algorithm that combines the basic technique of decision tree learning with the standard Logistic Regression functions at the leaves. The LogitBoost algorithm is used to fit iteratively the Logistic Regression at each node in the tree by using five-cross validation to determine the appropriate number of iteration while J48 is applied to each node for splitting. If the node is a nominal attribute; then, it will be split into k-value of child nodes, and if the node is a numeric attribute; then, it will be split into only two child nodes. These two nodes will be compared to a threshold; if the values of the instances is less than the value of the threshold; then, they will be sorted to the left side; otherwise, they will be sorted to the right side. The splitting will continue until the criterion is met.

Random Forest (RF) [27, 28] is a decision tree that follows the strategy of the ensemble method which combines more than one tree-structured classifier. Independent and random vectors are identically distributed among the structured trees. The grown trees are built randomly and mostly controlled by the generated random vectors. The accuracy of the classification prediction has significantly improved due to the algorithm of trees combination in which the most popular classes are selected based on the vote mechanism at the input *x* of vector.

### Dealing with imbalanced dataset

The five-year FIT Project dataset consists of 32,555 instances and composed of a heterogeneous sample of diabetic and non-diabetic patients. However, diabetic patients represent only 15.7% of the whole sample while non-diabetic patients represent 85.3%. The variance between the two classes is considerably large and could lead to lower accuracy on the prediction of the classifiers. In general, balance and imbalance classes are two representations of datasets. In most cases, the real-world data is imbalanced in many applications such as fraud detection, prevalence of diseases, credit scoring, or medical diagnosis. Class imbalance is a supervised learning problem and is very popular in the community of data science. The class imbalance problem occurs when there is a big difference between the number of majority class and the minority class and mostly in classes with binary values [29, 30]. The disparity caused in the values of the target class could have an extremely negative impact on the performance of the machine learning algorithms [31]. Most of the time, it would lead to false classification and the prediction result will be either over-fitted because the model does not attenuate the bias for the majority class or under-performed due to the very few instances of positive class [32].

In practice, several studies have shown that better prediction performance can be achieved by having balanced data; therefore, a number of well-known methods has been developed and used in machine learning to tackle this issue for improving the prediction models’ performance [33]. These methods [33–36] are called “Sampling Methods”. The main concept of these methods is to modify the original dataset target class values to equal the distribution in the label class. Under-sampling and over-sampling methods are applied in many forms. In our study, we designed our classification models experiments based on two techniques which are: Random Under-Sampling and Synthetic Minority Over-Sampling Technique (SMOTE). Previous studies showed that the random undersampling technique outperforms the SMOTE technique with some datasets; however, there are other studies that observed that SMOTE performed better with other datasets [37].

#### Random Under-Sampling technique

In this technique, all instances in the minority class are used while some instances of the majority class are removed randomly until both classes are equally balanced. One drawback of this technique is the loss of important information from the majority class. In this study, we used the undersampling method in three experiments by changing the values of the distribution spread in three levels (1.00, 1.50, and 2.00). As a result, three new training datasets are generated. Random Under-Sampling with (2.00) distribution spread value decreased the majority sample from 27,456 patients to 12,747 patients. Random Under-Sampling with (1.50) distribution spread value decreased the majority sample to 7,648 patients while with (1.00) distribution spread value, the majority sample has been decreased to 5,099 patients which is equal to the number of the minority class of positive cases (Table 2).

**Table 2:** Number of instances decreased by Random Under-Sampling technique.

Distribution Spread	Class “No” Actual 27456 (84.3%)	Class “Yes” Actual 5099 (15.7%)
2.50	12747	5099
1.50	7648	5099
1.00	5099	5099

#### Synthetic Minority Oversampling Technique (SMOTE)

The SMOTE technique is a type of oversampling method that has been shown to be powerful and is widely used in machine learning with imbalance high-dimensional data that are increasingly used in medicine [37]. The SMOTE technique generates randomly new examples or instances of the minority class from the nearest neighbors of line joining the minority class sample to increase the number of instances. These instances are created based on the features of the original dataset so that they become similar to the original instances of the minority class [38]. In our study, we applied the SMOTE techniques with three different percentages: 100%, 200%, and 300%. As a result, three new training datasets were generated. SMOTE with (100%) increased the positive sample from 5,099 instances of the minority class to 10,198 patients. SMOTE with (200%) increased the positive sample from 5,099 to 15,297 instances. SMOTE with (300%) increased the positive sample from 5,099 to 20,396 instances. This made an incremental increase in the minority class from 15.7% in the original dataset to 47% in the SMOTE with 300% dataset (Table 3).

**Table 3:** Number of instances increased by SMOTE technique.

Percentage of SMOTE Increase	Class “No” Actual 27456 (84.3%)	Class “Yes” Actual 5099 (15.7%)
100%	27456	10198
200%	27456	15297
300%	27456	20396

### Model validation

In general, there are two main validation methods used, namely the Hold-out method and K-fold Cross Validation method, in machine learning to validate the model’s performance after training the classifier [39–42]. The selection of each method depends on the goal of each classification problem and the data size. The Hold-out method divides the dataset into two data sets, training and test. The training set will be used to train the algorithm and will be evaluated against the test set which is the unseen data. The K-fold Cross Validation method [42], which we have used in this study, uses the whole dataset to be trained and tested by the given algorithm. First, the dataset is separated into *K* parts called folds, and all the folds have instances of equal size. The training process is applied on all folds except one fold for testing. This process is iterative and is repeated by the specified *K* number, where each fold has the chance to be tested once. The final performance measure will be the average of all the tests performance of all folds. The advantage of this method is that all the instances of the whole dataset are trained and tested, so lower variance occurs within the set estimator. This ensures a more accurate prediction and less bias of the true rate estimator; however, this method is computationally intensive and the validation takes a long time to be completed. In our study, we have relied on the 10-fold Cross Validation method, which has been used in several health care and medical related studies [43, 44].

## Results

**Fig 1:**
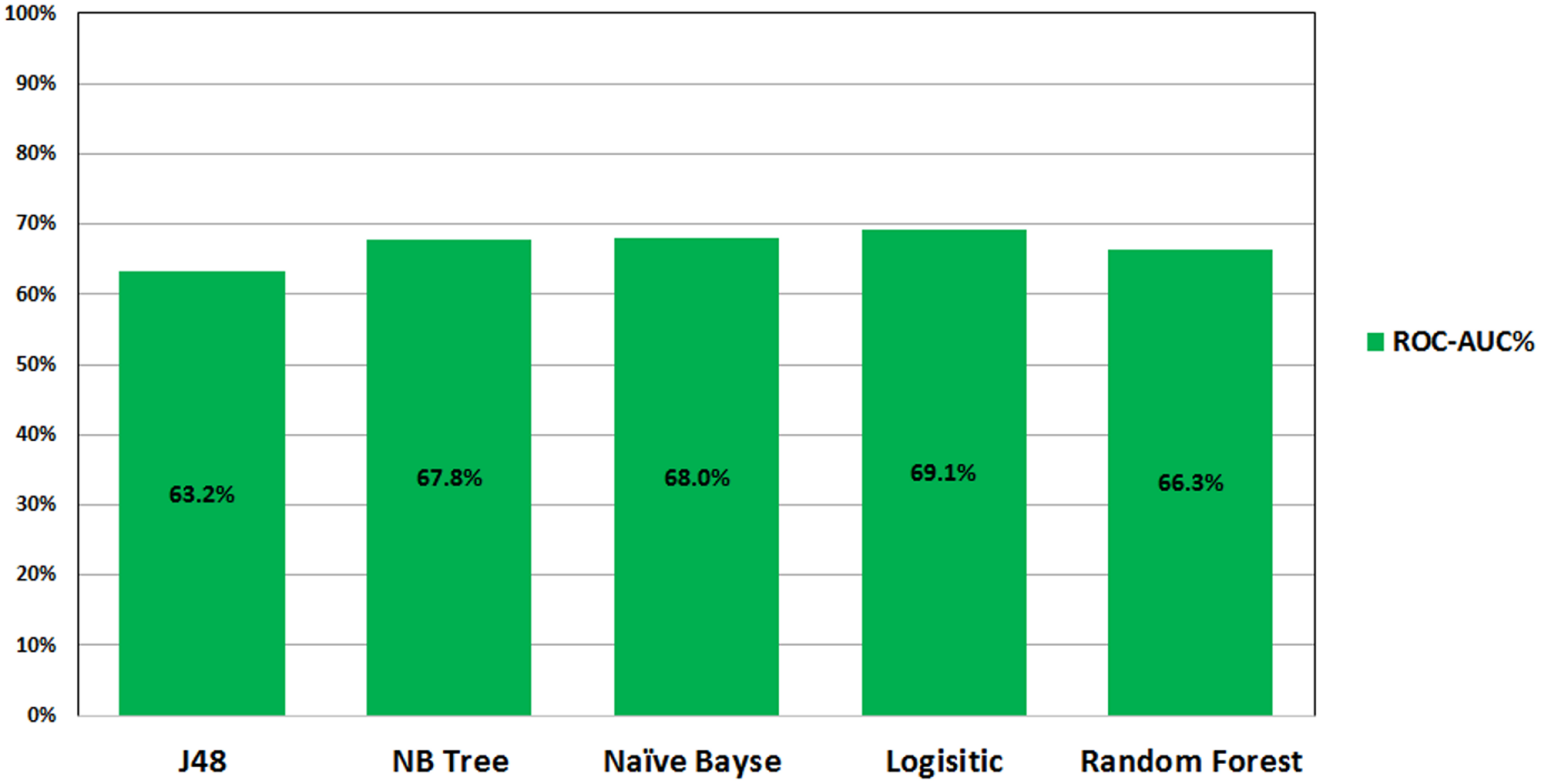
ROC performance of classification models on imbalance dataset using G1.

**Fig 2:**
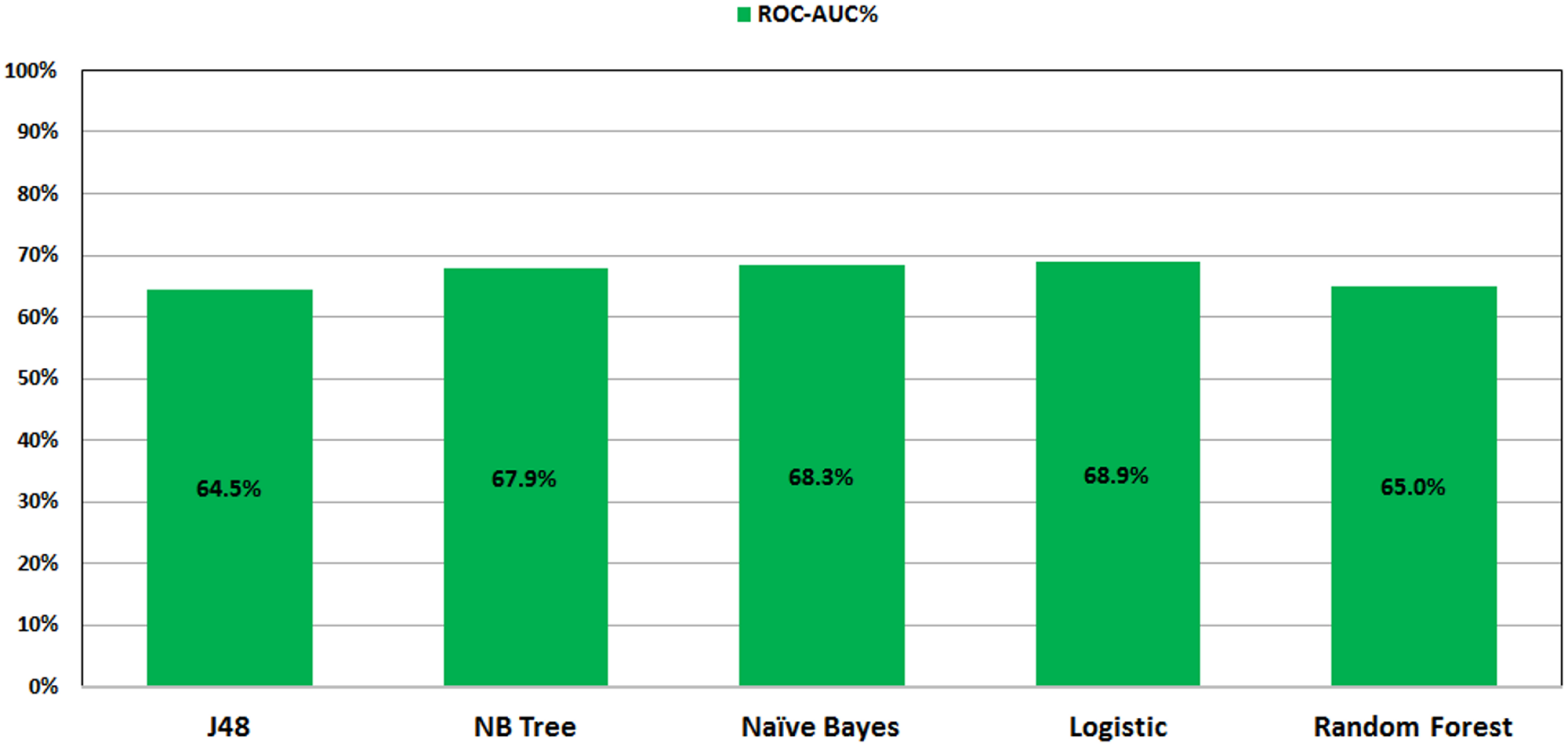
ROC performance of classification models on imbalance dataset using G2.

Figs 1 and 2 show the ROC performance of the ML classification methods on the imbalanced datasets using the two sets of attributes: G1 and G2, respectively. The results show that the Logistic Regression (LR) classifier achieves the highest performance (69.1% for G1 and 68.9% for G2) while the J48 Decision Tree (DT) classifier achieves the lowest performance (63.2% for G1 and 64.5% for G2).

Tables 4 and 5 show the detailed performance results of the ML classification methods on the imbalanced datasets using the two sets of attributes G1 and G2, respectively. With G1 attributes, the Naïve Bayes (NB) classifier achieves the best performance of the Kappa (15.4), Specificity (27.7%) and Precision (86.7%) metrics. The Random Forest (RF) classifier achieves the best performance of the Recall (99.9%) and Accuracy (84.3%). The Random Forest (RF) and the Logistic Regression (LR) classifiers jointly achieve the highest F1-Score (91.5). With G2 attributes, the Logistic Model Tree (LMT) classifier achieves the best performance of the Kappa (3.63) metric. The Naïve Bayes (NB) classifier achieves the best performance of the Specificity (21.2%) and Precision (86.1%). The Logistic Regression (LR) achieves the highest F1-Score (91.5).

**Table 4:** Evaluation of the performance of classification models on imbalance dataset using the G1 attributes.

Model	Kappa	Recall (%)	Specificity (%)	Precision (%)	Accuracy (%)	F1-Score
**J48**	2.45	98.5	3.0	84.5	83.58	91
**LMT**	5.93	98.1	5.8	84.9	83.64	91
**NB**	(15.4)	87.4	(27.7)	(86.7)	78.8	87.1
**LR**	0.92	99.8	0.70	84.4	84.29	(91.5)
**RF**	00.7	(99.9)	00.6	84.4	(84.3)	(91.5)

**Table 5:** Evaluation of the performance of classification models on imbalance dataset using the G2 attributes.

Model	Kappa	Recall (%)	Specificity (%)	Precision (%)	Accuracy (%)	F1-Score
**J48**	1.34	99.2	1.6	8.44	83.93	91.2
**LMT**	(3.63)	99.2	3.1	84.6	84.14	91.3
**NB**	1.37	90.8	(21.2)	(86.1)	79.94	88.4
**LR**	0.70	(99.9)	0.50	84.4	84.32	(91.5)
**RF**	1.14	99.4	1.3	84.4	84.04	91.3

**Fig 3:**
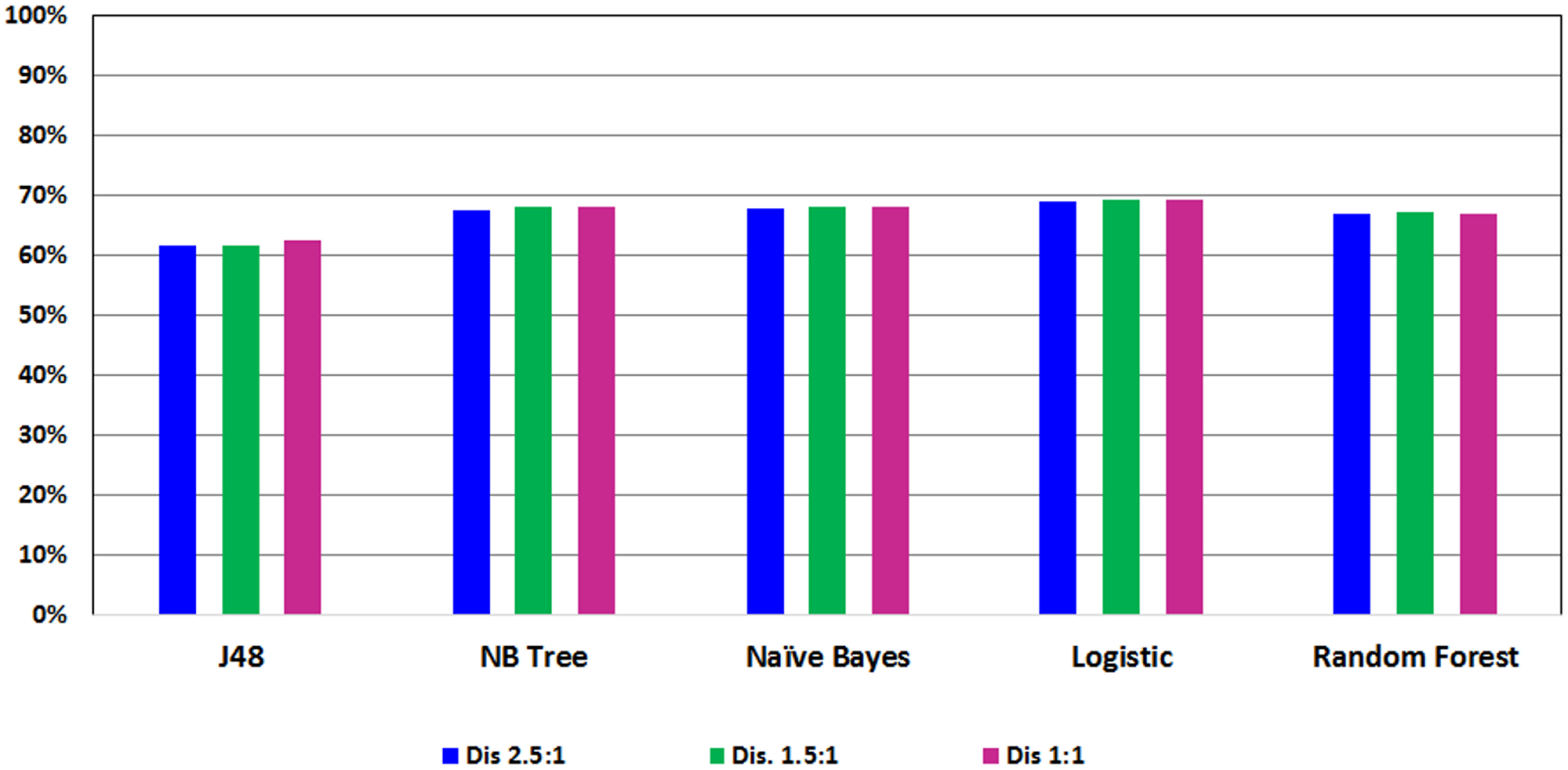
Performance of classification models on balance dataset using Random Under-Sampling.

**Fig 4:**
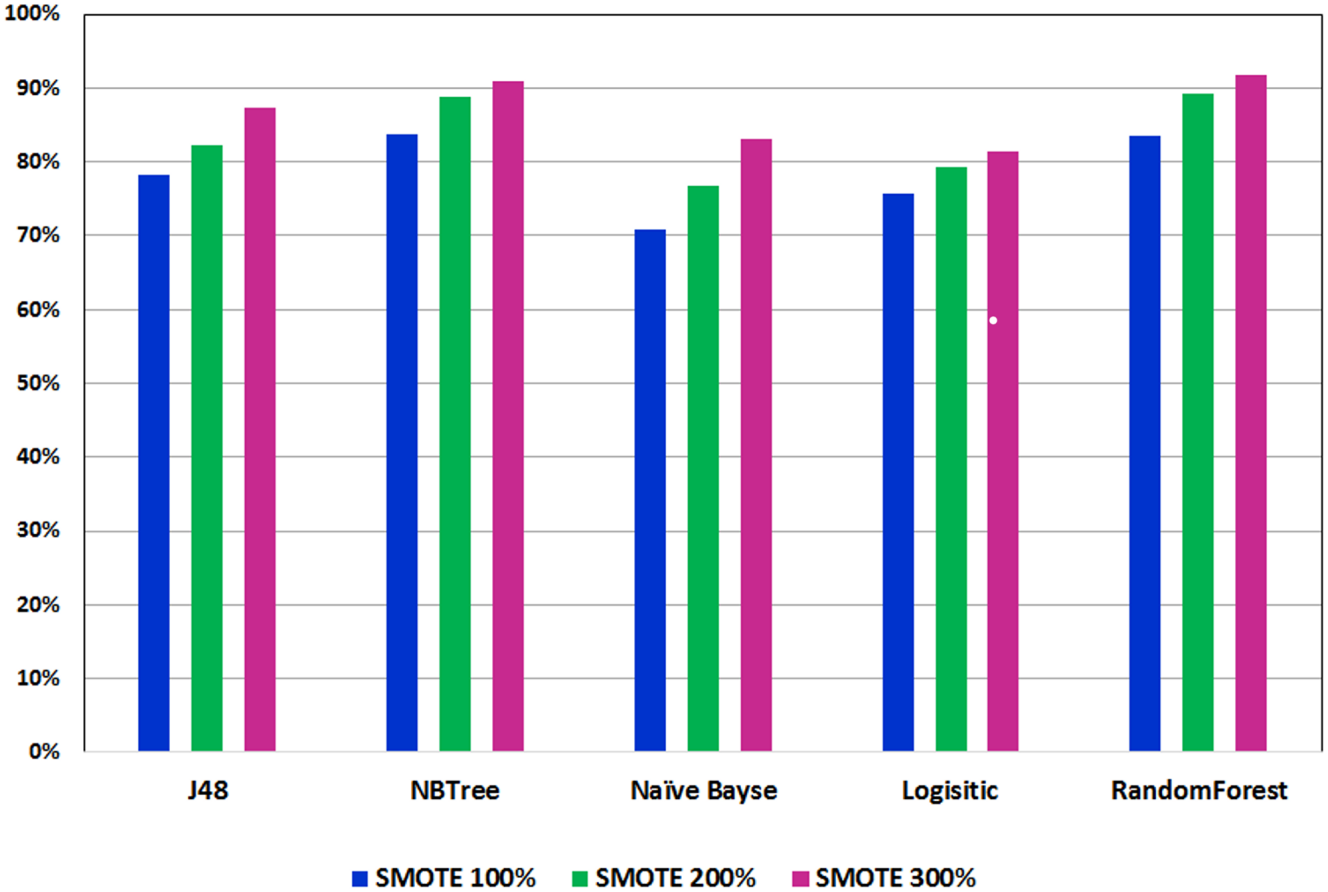
Performance of classification models on balance dataset using SMOTE.

Figs 3 and 4 show the ROC performance of the ML classification methods with the G2 attributes using the balanced datasets which are generated using the two sampling methods: Random Under-Sampling technique and SMOTE techniques, respectively. The results show that the Random Under-Sampling did not effectively the ROC performance of the classification models. With this sampling method, the Logistic Regression (LR) classifier achieved the highest ROC performance for distribution spread 1 (69.1%), distribution spread 1.5 (69.1%) and distribution spread 2.5 (68.8%). On the other hand, the results show that the SMOTE technique has effectively improved the ROC performance of the classification models. In particular, the Logistic Model Tree (LMT) achieved the highest ROC performance for the 100% increase (83.7%) and the 200% increase (88.9%) while the Random Forests (RF) classifier achieved the highest ROC performance for the 300% increase (91.8%). With the SMOTE technique, the ROC performance improve the sampling percentage increased.

In general, the ensemble learning approach applies the concept of collecting multiple individual classifiers and combines their predictions into one decision classifier [45]. The ensembling technique in machine learning has shown to be very efficient in improving the classification accuracy [18]. For example, Liu et al. [46] presented a method called *iDNA-KACC* which combines the support vector machine (SVM) and the auto-cross covariance transformation to identify the DNA-binding proteins only based on the protein sequence information. Liu et al. [47] has also presented another ensemble learning framework, called *iDHS-EL*, for identifying the location of DHS in human genome by fusing three individual Random Forest (RF) classifiers into an ensemble predictor. *iRSpot-EL* [48] is another ensemble learning framework which has been designed to identify recombination spots by fusing different modes of pseudo K-tuple nucleotide composition and mode of dinucleotide-based auto-cross covariance. Song et al. [49] employed an ensemble classifier using a new predictor (nDNA-Prot) to obtain the protein structure and identify DNA-binding proteins. The identification was conducted using a feature that selected the minimum Redundancy and Maximum Relevance (mRMR). Wang et al. [50] used an ensemble learning concept in combination with weights and sample misclassification information to effectively classify imbalanced data.

Table 6 shows the results of the Ensembling “Vote” method with three Decision Trees (Naiive Bayes, Random Forest, and Logistic Model Tree) on improving the overall ROC performance of the model classifiers to 92.2%. In particular, the ROC performance has increased by (0.4%) over the best ROC performance achieved by the Random Forests (RF) classifier (91.8%). The results of Table 6 show also that the ROC performance of the Ensembling “Vote” method using the set of attributes (G1) is very comparable to the ROC performance using the set of attributes (G2).

**Table 6:** Evaluation of the performance of classification models on imbalance dataset using the G2 attributes.

	ROC	Kappa	Recall (%)	Specificity (%)	Precision (%)	Accuracy (%)	F1-Score
**G1**	92.2	76.8	99.7	74.7	84.1	89.0	91.3
**G2**	92.2	77	99.9	74.6	84.1	89.0	91.3

## Discussion

To the best of our knowledge, this is the first study for predicting incident diabetes using machine learning methods based on cardiorespiratory fitness data. This study take advantage of the unique opportunity provided by our access to a large and rich clinical research dataset of the FIT project. In this study, a combination of three decision tree models (Random Forest, NB Tree, and LMT) in the Ensembling “Vote” approach achieved a high accuracy prediction (AUC = 0.92) using 13 features. The features are age, resting heart rate, metabolic equivalent level, resting systolic blood pressure, resting diastolic blood pressure, sedentary lifestyle, black, obesity, hypertension, percentage of heart rate achieved, history of hyperlipidemia, use of aspirin medication and family history of premature coronary artery disease.

With accelerating economic growth and changing lifestyles worldwide, it is important to evaluate and build predictive models for diabetes using common risk factors. Recently, machine learning methods have become of great interest and have been used by many scholars to build and compare models for predicting diseases including diabetes [20, 45]. For example, decision tree models have been widely used to predict diabetes [18] and experimental results showed that the weighted voting method not only improves the classification accuracy, but also has a strong generalization ability and universality [51, 52].

The prediction model developed by Habibi et. al. [4] used decision tree for screening T2DM which did not require laboratory tests for T2DM diagnosis. The prediction model is designed to identify T2DM patients and healthy people (AUC = 0.717) using 22,398 records. The model was built based on diagnosis variables defined by other studies as main predictor variables (age, Body Mass Index (BMI)) while sex, systolic and diastolic blood pressure, and family history of diabetes were found to be the highest risk factors.

Three machine learning models (logistic regression, artificial neural network, and decision tree) were used by Meng et. al. [18] for predicting diabetes and pre-diabetes based on 12 risk factors and a dataset of 1,487 patients. The results obtained from the comparison among these three models was in terms of their accuracy, sensitivity, and specificity; the best accuracy achieved was by using the decision tree model (77.87%) followed by the logistic regression model (76.13%), and finally the ANN (73.23%). The increase of age, family history of diabetes, BMI, and preference for salty food increases a person’s risk of developing diabetes while education level and drinking coffee showed a negative relationship with the disease.

Farran et al. [53] built a model to predict the incidents of diabetes, hypertension, and comorbidity through applying machine-learning algorithms on a dataset of 13,647,408 medical records for various ethnicities in Kuwait. The result of the classification accuracy for the four techniques was relatively high (80.7%) for Logistic Regression (LR), 78.6% for K-Nearest Neighbors (KNN), 78.30% for Multi-factor Dimensionality Reduction (MDR), 81.3% for Support Vector Machines (SVM), and 82% represents the result for the performance of all techniques collaboratively. The used models show that ethnicity is a significant factor for predicting diabetes.

In general, machine learning methods can provide great support for healthcare systems in various ways such as managing the hospital resources, recognizing high-risk patients, ranking the hospital, and improving patient care [54]. Healthcare organizations should leverage the advantage provided by machine learning tools to reduce the expenses of diabetes incidents by preventing the occurrence of the disease; thus, improving the public health and the population in general. However, one of the biggest challenges of machine learning in healthcare is that of data quality and consistency. Small dataset size, low quality of the data, incomplete data, and the lack of standardizations and interoperability may negatively affect the ability of building models that provide effective prediction.

## Conclusion

Although a large body of research efforts has accumulated to design methods that can predict incident diabetes, the majority of these methods uses traditional statistical methods. Machine learning methods are increasingly gaining momentum and the attention of the healthcare community. This study shows the potential of machine learning methods for predicting incident diabetes using cardiorespiratory fitness data. We have investigated 42 demographic and clinical features for (32,555) patients of the FIT Project who were non-diabetic at baseline and took the stress mill test; then they were followed up for five years. Applying the Random Under-Sampling technique showed no improvement on the five classification models used in this study. On the other hand, the SMOTE technique showed significant improvement on the prediction of all classification models prediction performance in line with the gradual increase of the percentages used. The Random Forest and NB Tree models showed greater results in all model evaluation metrics (Kappa, Recall, Precision and Specificity). The two models achieved AUC of 0.916 and 0.917, respectively. In order to further enhance the the prediction accuracy, we used an ensembling method, specifically with the “Vote” technique that combined three decision tree classification methods (Random Forest, NB Tree, and LMT). The ensembling method improved the prediction accuracy to AUC = 0.922. The study shows the potential of ensembling and SMOTE approaches for predicting incident diabetes using cardiorespiratory fitness data. In general, our results significantly outperform the results which are reported in other reports of the literature. However, more work can be done to further increase the quality of prediction by exploring other machine learning models. In our future work, we will for validating our results with other related cohorts.

## Ethical approval

This article does not contain any studies with human participants or animals performed by any of the authors. The FIT project is approved by the IRB (ethics committee) of HFH hospital (IRB #: 5812).

## Supporting information

S1 Appendix Description of the final dataset. (DOCX)
