# Supplementary material for: Predicting diabetes mellitus using SMOTE and ensemble machine learning approach: The Henry Ford ExercIse Testing (FIT) project
Source: PLoS One. 2017 Jul 24;12(7):e0179805. doi: 10.1371/journal.pone.0179805 (PMC5524285; doi:10.1371/journal.pone.0179805)
Supplement: S1 Appendix — (DOCX) [file pone.0179805.s001.docx]

**Appendix A: Description of the Final Dataset**

| **Attributes** | **Class** | | | | | | | **Pearson Correlation** | **p-value** | | **Mean** | | | **SD.** | |
| --- | --- | --- | --- | --- | --- | --- | --- | --- | --- | --- | --- | --- | --- | --- | --- |
|  | **Diabetic**  27456  84.3% | **Non-Diabetic**  5099  15.7% | | | | **Total**  32555 | |  |  |  |  |  |  |  |  |
| ***Demographic Characteristic*** | | | | | | | | | | | | | | | |
| *sex* | Binary | | Male  18093 (55.6%) | Female  14462  (44.4%) | | | .024 | | .000 | | | 1.44 | | | .497 |
| *black* | Binary | | No  23691  (72.8%) | Yes  8864  (27.2%) | | | .067 | | .000 | | | .27 | | | .445 |
| *target heart rate*  *0 - 131.8*  *131.8 - 139.6*  *139.6 – 147.4*  *147.4 – 155.2*  *155.2 – 163*  *163 – 170.8*  *170.8 – 178.6*  *178.6 – 186.4*  *186.4 – 194.2*  *194.2>* | Nominal | | 15  385  2155  4178  5620  7136  7420  3923  1366  357 | 0%  1%  6.6%  13%  17%  23%  23%  12%  4%  1% | | | -.139- | | .000 | | | 166.69 | | | 12.634 |
| ***Stress Test Result*** | | | | | | | | | | | | | | | |
| *MET Level*  *1*  *2*  *3*  *4* | Nominal | 4391  8314  11640  8210 | | | 13%  26%  36%  25% | | -.177 | | .000 | | | 9.26 | | | 3.088 |
| *resting heart rate*  *<78*  *>78* | Numeric | 22896  9659 | | | 70%  30% | | .031 | | .000 | | | 72.49 | | | 12.573 |
| *resting systolic BP*  *<=99.6*  *99 – 119*  *119 – 139*  *139 -158*  *158 – 178*  *>=178* | Numeric | 822  7562  13391  7775  2548  457 | | | 2.5%  23%  41%  23.8%  7.8%  1.4% | | .091 | | .001 | | | 130.76 | | | 19.034 |
| *resting diastolic BP*  *<60*  *60 – 71*  *71 – 82*  *82 – 92*  *92 – 103*  *>103* | Numeric | 1360  5009  12556  9612  3355  663 | | | 4%  15.3%  39%  30%  10.3  2% | | .037 | | .186 | | | 81.55 | | | 10.609 |
| *Percent heart achieved*  *<.67*  *.67 – 1*  *>1* | Numeric | 1437  30863  255 | | | 4.4%  94.8%  .7% | | -.084- | | .000 | | | .90041 | | | .11030 |
| ***Medication Use*** | | | | | | | | | | | | | | | |
| *Aspirin* | Binary | No | | | | Yes | | .038 | .261 | .22 | | | .412 | | |
|  |  | 25490  (78.3%) | | | | 7065  (21.7%) | |  |  |  |  |  |  |  |  |
| *Palvix* | Binary | 32012  (98.3%) | | | | 543  (1.7%) | | .026 | .339 | .02 | | | .128 | | |
| *Beta Blocker* | Binary | 26001  (79.9%) | | | | 6554  (20%) | | .089 | .000 | .20 | | | .401 | | |
| *Angiotensin Converting Enzyme inhibitor* | Binary | 28052  (86.2%) | | | | 4503  (13.8%) | | .095 | .000 | .14 | | | .345 | | |
| *Angiotensin Receptor Blockers* | Binary | 32029  (98.4%) | | | | 526  (1.6%) | | .031 | .872 | .02 | | | .126 | | |
| *Nitrate* | Binary | 29867  (91.7%) | | | | 2688  (8.3%) | | .025 | .000 | .08 | | | .275 | | |
| *Diuretic* | Binary | 27307  (83.9%) | | | | 5248  (16%) | | .129 | .000 | .16 | | | .368 | | |
| *Calcium Channel Blocker* | Binary | 28462  (87.4%) | | | | 4093  (12.6%) | | .064 | .865 | .13 | | | .332 | | |
| *Other Hypertension Medication Use* | Binary | 31523  (96.8%) | | | | 1032  (3.2%) | | .051 | .017 | .03 | | | .175 | | |
| *History and Patient Status* | | | | | | | | | | | | | | | |
| *Family history of premature coronary artery disease* | Binary | 15742  (48.4%) | | | | 16813  (51.6%) | | -.012- | .825 | .52 | | | .500 | | |
| *Obesity* | Binary | 6048  (18.6%) | | | | 26507  (81.4%) | | .106 | .000 | .19 | | | .389 | | |
| *Smoke* | Binary | 18865  (57.9%) | | | | 13690  (42.1%) | | .012 | .496 | .42 | | | .494 | | |
| *Sedentary Lifestyle* | Binary | 22348  (68.6%) | | | | 10207  (31.4%) | | .053 | .000 | .31 | | | .464 | | |
| ***Diseases*** | | | | | | | | | | | | | | | |
| *Hypertension* | Binary | 12964  (39.8%) | | | | 19591  (60.2%) | | .148 | .000 | .60 | | | .490 | | |
| *Prior cerebrovascular accident* | Binary | 32009  (98.3%) | | | | 546  (1.7%) | | .043 | .001 | .02 | | | .128 | | |
| *Hyperlipidemia* | Binary | 19110  (58.7%) | | | | 13445  (41.3%) | | .065 | .000 | .41 | | | .492 | | |
| *Coronary Artery Disease* | Binary | 27939  (85.8%) | | | | 4616  (14.8%) | | .045 | .118 | .14 | | | .349 | | |
| *Congestive Heart Failure* | Binary | 31847  (97.8) | | | | 708  (2.2%) | | .055 | .000 | .02 | | | .146 | | |
